# Supplementary material for: Iterative improvement in the automatic modular design of robot swarms
Source: PeerJ Comput Sci. 2020 Dec 7;6:e322. doi: 10.7717/peerj-cs.322 (PMC7924708; doi:10.7717/peerj-cs.322)
Supplement: Supplemental Information 3 [file peerj-cs-06-322-s003.zip › argos3/doc/api/standalone/a00644_source.html]

ARGoS: plugins/simulator/visualizations/qt-opengl/qtopengl\_lua\_find\_dialog.cpp Source File


- Main Page
- Related Pages
- Namespaces
- Classes
- Files

- File List
- File Members

# plugins/simulator/visualizations/qt-opengl/qtopengl\_lua\_find\_dialog.cpp

Go to the documentation of this file.

```
00001 
00007 #include "qtopengl_lua_find_dialog.h"
00008 
00009 #include <QCheckBox>
00010 #include <QDialogButtonBox>
00011 #include <QGroupBox>
00012 #include <QHBoxLayout>
00013 #include <QLabel>
00014 #include <QLineEdit>
00015 #include <QPushButton>
00016 #include <QVBoxLayout>
00017 
00018 namespace argos {
00019 
00020    /****************************************/
00021    /****************************************/
00022 
00023    CQTOpenGLLuaFindDialog::CQTOpenGLLuaFindDialog(QWidget *parent) :
00024       QDialog(parent) {
00025       m_pcLabel = new QLabel(tr("&Find text:"));
00026       m_pcLineEdit = new QLineEdit();
00027       m_pcLabel->setBuddy(m_pcLineEdit);
00028 
00029       m_pcCaseCheckBox = new QCheckBox(tr("Match &case"));
00030       m_pcFromStartCheckBox = new QCheckBox(tr("Search from &start"));
00031       m_pcFromStartCheckBox->setChecked(true);
00032 
00033       m_pcFindButton = new QPushButton(tr("&Find"));
00034       m_pcFindButton->setDefault(true);
00035 
00036       m_pcMoreButton = new QPushButton(tr("&More"));
00037       m_pcMoreButton->setCheckable(true);
00038       m_pcMoreButton->setAutoDefault(false);
00039 
00040       m_pcButtonBox = new QDialogButtonBox(Qt::Vertical);
00041       m_pcButtonBox->addButton(m_pcFindButton, QDialogButtonBox::ActionRole);
00042       m_pcButtonBox->addButton(m_pcMoreButton, QDialogButtonBox::ActionRole);
00043 
00044       m_pcExtension = new QWidget();
00045 
00046       m_pcWholeWordsCheckBox = new QCheckBox(tr("&Whole words"));
00047       m_pcBackwardCheckBox = new QCheckBox(tr("Search &backward"));
00048       m_pcSearchSelectionCheckBox = new QCheckBox(tr("Search se&lection"));
00049 
00050       connect(m_pcMoreButton, SIGNAL(toggled(bool)),
00051               m_pcExtension, SLOT(setVisible(bool)));
00052 
00053       QVBoxLayout* m_pcExtensionLayout = new QVBoxLayout();
00054       m_pcExtensionLayout->setMargin(0);
00055       m_pcExtensionLayout->addWidget(m_pcWholeWordsCheckBox);
00056       m_pcExtensionLayout->addWidget(m_pcBackwardCheckBox);
00057       m_pcExtensionLayout->addWidget(m_pcSearchSelectionCheckBox);
00058       m_pcExtension->setLayout(m_pcExtensionLayout);
00059 
00060       QHBoxLayout* m_pcTopLeftLayout = new QHBoxLayout();
00061       m_pcTopLeftLayout->addWidget(m_pcLabel);
00062       m_pcTopLeftLayout->addWidget(m_pcLineEdit);
00063 
00064       QVBoxLayout* m_pcLeftLayout = new QVBoxLayout();
00065       m_pcLeftLayout->addLayout(m_pcTopLeftLayout);
00066       m_pcLeftLayout->addWidget(m_pcCaseCheckBox);
00067       m_pcLeftLayout->addWidget(m_pcFromStartCheckBox);
00068       m_pcLeftLayout->addStretch(1);
00069 
00070       QGridLayout* m_pcMainLayout = new QGridLayout();
00071       m_pcMainLayout->setSizeConstraint(QLayout::SetFixedSize);
00072       m_pcMainLayout->addLayout(m_pcLeftLayout, 0, 0);
00073       m_pcMainLayout->addWidget(m_pcButtonBox, 0, 1);
00074       m_pcMainLayout->addWidget(m_pcExtension, 1, 0, 1, 2);
00075       setLayout(m_pcMainLayout);
00076 
00077       setWindowTitle(tr("Find/replace"));
00078       m_pcExtension->hide();
00079    }
00080 
00081    /****************************************/
00082    /****************************************/
00083 
00084 }
```

---

Generated on 10 Jul 2018 for ARGoS by 
 1.6.1
